# Outcome of repeat surgery for genital prolapse using prolift-mesh

**DOI:** 10.1186/1750-1164-7-3

**Published:** 2013-03-05

**Authors:** Ibrahim A Yakasai, Lawal A Bappa, Andrew Paterson

**Affiliations:** 1 Departments of Obstetrics and Gynaecology, Bayero University/Aminu Kano teaching Hospital, Kano, Nigeria; 2 Doncaster Royal Infirmary and Royal Alexandra Hospital, Paisley, United Kingdom; 3 Department of Obstetrics and Gynaecology, Aminu Kano Teaching Hospital Kano, Kano, PMB3452, Nigeria

**Keywords:** Prolift, Mesh, Urogenital prolapse

## Abstract

**Introduction:**

Urogenital prolapse can have a significant impact on quality of life. The life time risk of requiring surgery for urogenital prolapse is 11%. Prolift mesh has recently been introduced to reduce repeat operation rate and for long-term benefit.

**Objective:**

To evaluate the outcome of the treatment of urogenital prolapse with synthetic mesh.

**Methods:**

A retrospective review of case notes of all women who underwent prolift mesh insertion for prolapse between July 2004 and June 2005, at Royal Alexandra Hospital Paisley UK. We looked at the presenting complaints, previous operation, intraoperative complications and complications at six weeks and six months follow-up.

**Results:**

Twenty-two procedures were carried out in the twelve months period. Age of the patients ranged from 55 to 82 years (median 64 yrs). Eleven had anterior Prolift (50%), Seven had posterior Prolift 31.8% and four total Prolift 18%. There were no intraoperative complications. All the patients had previous surgery for prolapse. Eight patients had anterior repair, six patients had posterior repair, and three patients had abdominal hysterectomy. Vaginal hysterectomy was carried out with mesh insertion as a concomitant procedure in seven cases (31.25%). All patients were seen at six weeks and six months after the surgery. Complications rate included mesh erosion one patient and suture material protruding in the vagina one patient, one patient had failed prolift operation. All the twenty-one patients were cured giving 95.4% success rate.

**Conclusion:**

The use of prolene mesh in pelvic reconstructive surgery was associated with good outcome and minimal complications in this study.

## Background

Genital Prolapse surgery has varying degree of success. Synthetic meshes are increasingly used in the surgical management of pelvic organ prolapse in an attempt to improve the success rates and to increase longevity of repairs.

If there are no urinary symptoms urodynamics studies are not justified outside the research setting. Surgically the key issues are which technique produces the best; long-lasting anatomical result. There is no widely accepted and standardized technique for the management of recurrent prolapse. Multiple surgical techniques have evolved each supported enthusiastically by their proponents and some of the techniques involve the use of synthetic mesh material.

## Methods

A retrospective review of all case notes of women who had repeat prolapse operation with mesh at Royal Alexandra Hospital Paisley, U.K, between March 2005 and Feb 2006 was carried out. All women had a standardized urogynaecological history and examination performed before and after the surgery, including that on presenting complaints, previous operation, intraoperational complication and complication at 6 weeks and 6 month follow-up. For anterior Prolift a midline incision was made along the anterior vaginal wallsuburetherally to the vaginal apex and the bladder was reflected from the vagina. This dissection was extended bilaterally to the ischial spines and advanced anteriorly along the arcus tendineus. Midline placation of the fascial layer was performed using interrupted 2/0 polydioxanon (PDS). Atrium mesh (2×15 cm) with a widened elliptical midportion was placed under the bladder base and each lateral extension was positioned on to the iliococcygeal fascia anterior to the ischial spines. The mesh overlay was sutured with 2/0 Polyglactin (vicryl) sutures at the anterior and posterior margins to prevent it folding (Table 1).

**Table 1:** TYPES OF MESH REPAIR

**Type of operation**	**Number (%)**
**Anterior mesh**	**11 (50%)**
**Posterior mesh**	**7 (31%)**
**Anterior and posterior**	**4 (18%)**

We carry out the posterior compartment mesh repair by using a midline incision from the perineum to the vaginal apex and the vagina detached from the rectum with a sharp dissection, which was extended laterally to the ischiorectal fossa, and superiorly onto the sacrospinous ligament. Fascial defect in the rectovaginal septum was repaired using 2/0 polydioxanon (PDS) interrupted sutures. Atrium mesh 10×15 cm was fashioned in a Y-shape, the arms of the Y, 2 cm wide and the body 5 cm wide. The arms of the Y were placed onto the sacrospinous ligament bilaterally with the main body of mesh overlaying the repaired rectovaginal fascia and the perineal body. The mesh was also stabilized with vicryl 2/0 sutures placed superiorly. And laterally onto the perineal body. We routinely performed rectal examination was in order to exclude damage or inadvertent placement of the sutures in the rectum.

Following placement of the mesh overlay the vagina was closed a cystoscopy and rectal examination was performed to exclude any urinary or rectal injury.

## Results

Twenty-two procedures were carried out in the twelve months period. Age of the patients ranged from 55 to 82 years (median 64 yrs). Eleven had anterior Prolift (50%), Seven were posterior Prolift 31.8% and Four total Prolift 18%. There were no intra operative complications. All the patients had previous surgery for prolapse. Eight patients had anterior repair, Six patients had posterior repair, and three patients had abdominal hysterectomy. Vaginal hysterectomy was carried out with mesh insertion as a concomitant procedure in seven cases (31.25%). All patients were seen at Six weeks and six months after the surgery. Complications rate included mesh erosion one patient and suture material protruding in the vagina one patient at the six months follow-up. One patient had failed total Prolift operation. All the twenty-one patients were cured giving 95.4% success rate*.*

## Discussion

This study reports on 22 women who had pelvic floor reconstruction with anterior, posterior or total mesh reinforcement. The overall cure rate at 6 weeks and 6months was 95.4%.

A study reports that [1] with total mesh one patient failed and the procedure had to be repeated similar to our own studies. The appearance of prolapse in a well supported compartment is an issue which occurs after all surgery for prolapse whether conventional or using mesh [2-4].

This finding seems to be comparable to what has been reported after sacrospinous colpopexy and given that the mesh is fixed through sacrospinous ligament bilaterally, may well be for the same reason.

One patient had concomitant vagina hysterectomy and posterior Prolift, while three patients had vaginal hysterectomy as a concomitant procedure and anterior Prolift. In all these cases the procedures were successful. The remaining three concomitant vaginal hysterectomies were in the total prolift group and among them an elderly lady 82 years had a failed total Prolift. This probably may well be because of her age that predisposes to significant supporting ligaments weaknesses. Our success rate of 95.4% is comparable to most studies [5-7].

There are various type of mesh in the markets, however type 1monofilament polypropylene mesh with large pore sizes is currently recommended to reduce complications such as mesh erosion, extrusion inflammation or infection [8-10]. We use a similar type of mesh in our studies. This study reports 22 women who underwent pelvic floor reconstructions, with anterior, posterior or total mesh reinforcement. The overall cure rate at six months for all the three compartments respectively was 95.4%.

Concern continues to be voiced regarding the risk of chronic infection and the potentially disastrous consequences of mesh finding its way within a hollow viscus such as bladder [11-13]. We found only 2 instances of mesh erosion/protrusion in our follow-up patients and both were easily managed with excision of the protruding mesh resulting in complete cure.

Some studies have reported an up to 26% mesh erosion rate and up to 38% dyspareunia rate. We did not have dyspareunia as complication.

Most cases of prolapse in the Caucasian population is due to age factors that weaken the ligaments supporting the pelvic structures [14]. In this study the failure of the total mesh prolift was in the 82 year old lady. In an African population however most recorded cases of prolapse are due to the high parity and the patients are relatively younger. There is need to have a randomized trial to compare the effectiveness, of the various synthetic materials in our center in future. Cost is a prohibitive factor in purchasing the prolene mesh. It therefore implies in a low resource setting economy, especially the developing Nations, they will not be able to afford these prostheses for their practice.

## Conclusion

Our study confirms that mesh prolift procedure is safe in the hands of trained surgeons, with a success rate of 95.4% and minimal complication rate. Large randomized trials of conventional surgery versus mesh insertion will be necessary to answer major question on both the anatomical and functional outcome of pelvic floor repair.

## Competing interest

There is no financial interest in the information contained in this article.

## References

[B1] De Cuyper EM Frazer MI Pelvic organ prolapse repair with Proliftmesh: a prospective study Pelviperineology 2009 28 82 88

[B2] Marchionni M Bracco GL Checcucci V Carabaneanu A Coccia EM Mecacci F True incidence of vaginal vault prolapse. Thirteen years experience J Reprod Med 1999 44 679 684 10483537

[B3] Maher CF Qatawneh AM Dwyer PL Carey MP Cornish A Schluter PJ Abdominal sacral colpopexy or vaginal sacrospinous colpopexy for vaginal vault prolapse: a prospective randomized study Am J ObstetGynecol 2004 190 20 26 10.1016/j.ajog.2003.08.031 14749629

[B4] Hiltunen R Niemenen K Takala T Heiskanen E Merikari M Niemi K Low-weight polypropylene mesh fro anterior vaginal prolapse: a randomized controlled trial Obstet Gynecol 2007 50 455 462 17666627 10.1097/01.AOG.0000261899.87638.0a

[B5] Abdelfattah M Ramsay I West of Scotland Study G retrospective multicenter study of the new minimally invasive mesh repair devices for pelvic organ prolapse BJOG 2008 115 22 30 18053100 10.1111/j.1471-0528.2007.01558.x

[B6] Altman D falconer C Perioperative morbidity using Transvaginal mesh in pelvic floor organ prolapse repair Obstet Gynecol 2007 109 303 308 10.1097/01.AOG.0000250970.23128.63 17267828

[B7] Reisenaeur C Kirkschniak A Drews U Wallweiner D Anatomical conditions for pelvic floor reconstruction with polypropylene implant and its application for the treatment of vaginal prolapse Eur J Obstet Gynecol Reprod Biol 2007 131 214025 10.1016/j.ejogrb.2006.03.020 16677753

[B8] Luijendijik RW Hop WC MP v dT de Lange DC Braaksma MM Ijzermans JN A comparison of suture repair with mesh repair for incisional hernia N Engl J Med 2000 343 392 398 10.1056/NEJM200008103430603 10933738

[B9] Imparato E Aspesi G Rovetto E Presti M Surgical management and prevention of vaginal vault prolapse Surg Gynecol Obstet 1992 175 233 237 1514157

[B10] Welgoss JA Vogt VY McCalellan EJ Benson JT Relationship between surgically induced neuropathy and outcome of pelvic organ prolapse surgery Int Urogynecol J Pelvic Floor Dysfunct 1999 10 11 14 10.1007/PL00004007 10207761

[B11] Valatis SR Stanton SL Sacrocolpopexy: a retrospective study of clinician’s experience BJOG 1994 101 518 522 10.1111/j.1471-0528.1994.tb13154.x 8018642

[B12] Braizzolora S Pillai-Allen A Risk of mesh erosion with sacrocolpopexy and concurrent hysterectomy Am Coll Obstet Gynecol 2003 102 2 306 310 10.1016/S0029-7844(03)00515-5 PMC1364470 12907104

[B13] Sand PK Koduri S Lobel R Prospective randomized trial of polyglactin 910 mesh to prevent recurrence of cystocoele and rectocoele Am J Obstet Gynecol 2001 184 1357 1362 10.1067/mob.2001.115118 11408853

[B14] Dwyer PL Reilly BA Transvaginal repair of anterior and posterior compartment prolapse with atrium polypropylene mesh BJOG 2004 111 831 836 10.1111/j.1471-0528.2004.00194.x 15270932

